# Water, sanitation and hygiene infrastructure and quality in rural healthcare facilities in Rwanda

**DOI:** 10.1186/s12913-017-2460-4

**Published:** 2017-08-03

**Authors:** Alexandra Huttinger, Robert Dreibelbis, Felix Kayigamba, Fidel Ngabo, Leodomir Mfura, Brittney Merryweather, Amelie Cardon, Christine Moe

**Affiliations:** 1 The Center for Global Safe Water, Sanitation and Hygiene at Emory University, 1518 Clifton Rd. NE, Atlanta, GA 30322 USA; 2 London School of Hygiene and Tropical Medicine, Keppel St., London, WC1E 7HT UK; 3 The Access Project Rwanda, P.O. Box 7393, Kigali, Rwanda; 4 The Republic of Rwanda Ministry of Health Maternal and Child Health Unit, P.O. Box 84, Kigali, Rwanda

**Keywords:** Water, Sanitation, Hygiene, LMIC, Healthcare facilities, Policy implementation, Standards

## Abstract

**Background:**

WHO and UNICEF have proposed an action plan to achieve universal water, sanitation and hygiene (WASH) coverage in healthcare facilities (HCFs) by 2030. The WASH targets and indicators for HCFs include: an improved water source on the premises accessible to all users, basic sanitation facilities, a hand washing facility with soap and water at all sanitation facilities and patient care areas. To establish viable targets for WASH in HCFs, investigation beyond ‘access’ is needed to address the state of WASH infrastructure and service provision. Patient and caregiver use of WASH services is largely unaddressed in previous studies despite being critical for infection control.

**Methods:**

The state of WASH services used by staff, patients and caregivers was assessed in 17 rural HCFs in Rwanda. Site selection was non-random and predicated upon piped water and power supply. Direct observation and semi-structured interviews assessed drinking water treatment, presence and condition of sanitation facilities, provision of soap and water, and WASH-related maintenance and record keeping. Samples were collected from water sources and treated drinking water containers and analyzed for total coliforms, *E. coli*, and chlorine residual.

**Results:**

Drinking water treatment was reported at 15 of 17 sites. Three of 18 drinking water samples collected met the WHO guideline for free chlorine residual of >0.2 mg/l, 6 of 16 drinking water samples analyzed for total coliforms met the WHO guideline of <1 coliform/100 mL and 15 of 16 drinking water samples analyzed for *E. coli* met the WHO guideline of <1 *E. coli*/100 mL. HCF staff reported treating up to 20 L of drinking water per day. At all sites, 60% of water access points (160 of 267) were observed to be functional, 32% of hand washing locations (46 of 142) had water and soap and 44% of sanitary facilities (48 of 109) were in hygienic condition and accessible to patients. Regular maintenance of WASH infrastructure consisted of cleaning; no HCF had on-site capacity for performing repairs. Quarterly evaluations of HCFs for Rwanda’s Performance Based Financing system included WASH indicators.

**Conclusions:**

All HCFs met national policies for water access, but WHO guidelines for environmental standards including water quality were not fully satisfied. Access to WASH services at the HCFs differed between staff and patients and caregivers.

## Background

### Safe water in healthcare facilities

Healthcare facilities (HCFs) require adequate quantity and quality of water in order to maintain a hygienic environment. Improved sanitation, appropriate waste disposal and personal hygiene are all crucial. HCFs are recognized by the World Health Organization (WHO) as ‘environments with a high prevalence of infectious disease agents where patients, staff, carers and neighbors of the health-care setting face unacceptable risks of infection if environmental health is inadequate’ [1]. A systematic review of hospital-acquired infections (HAIs) in low- and middle-income countries concluded that inadequate environmental hygienic conditions were a substantial determinant of endemic HAIs infections [2]. WHO guidelines recommend that ‘health centers and hospitals should have consistent, or at least predictable, running water, clean toilets, safe refuse disposal, clean beds and areas for birthing’ [3]. Overcrowding and substandard infrastructure in HCFs in Africa compound risks for HAIs and may deter patients from coming to the HCF, particularly when hospitalization is required [4–7]. Guidelines for health care in emergency settings recommend that HCFs provide 40–60 L of water per patient per day and in non-emergency settings guidelines for environmental health in HCFs recommend an additional 5 L of water per outpatient per day [1]. Velleman et al. established a call to action for improving water sanitation and hygiene (WASH) for maternal and newborn health, emphasizing that improved WASH should be: 1) integrated into budget priorities for infrastructure and supplies, 2) emphasized in campaigns for maternal and child health, and 3) embedded into national and global targets and monitoring frameworks [8].

A 2012 UN Water report addressing water, sanitation, and hygiene in 74 countries (34 of which were in Africa) reported that an average of 13% of hospitals lacked improved water supply, defined as an improved water source within 500 m of the facility [9]. A 2015 report reviewed survey data on hospitals, health centers, and dispensaries in 51 low- and middle-income countries (21 of which were in Africa); the report demonstrated that, on average, 39% of HCFs did not have an improved water supply; and among HCFs in Africa that estimate was 47% [10]. These reports indicate that there is a gap in adequate water supply in HCFs in Africa, particularly in secondary HCFs. The severity of this problem is even more apparent when the availability of a reliable, year-round, on-site water supply is considered. Nationally representative surveys in five sub-Saharan African countries (Ghana, Kenya, Rwanda, Tanzania and Uganda), found that the percentage of hospitals (both government and private facilities) with dependable running water and power ranged from 22% in Tanzania to 46% in Rwanda [5]. The majority of available data focus on WASH in hospitals and there is very limited data available on the state of WASH in secondary HCFs, where the majority of healthcare services are delivered.

### Rural healthcare facilities in Rwanda

Rwanda is the most densely populated country in Africa (415 people/km^2^), and 80% of the population live in rural areas [11]. Rwanda has made exemplary progress in health system strengthening and reduction of preventable deaths. Since the 2000’s, maternal mortality has decreased 60%, mortality in children under 5 years of age has decreased 63%, and deaths from HIV, TB and malaria have decreased 80% [12–14]. Despite the detrimental effects of the 1994 genocide, prioritization of pro-poor economic development and investment in health system strengthening has resulted remarkable gains in population health: under five mortality in Rwanda is half the average for sub-Saharan Africa region despite the fact that Rwanda’s GDP per capita is two and a half times smaller than the regional average [15, 16]. Primary care in rural areas is delivered by health centers. There are 465 health centers in Rwanda each serving an average catchment population of approximately 20,000 people [11]. These facilities typically receive about 100 patients per day for services including basic emergency care, antenatal care, normal delivery, post-partum care, family planning, pediatric care and nutrition, and routine clinics [17]. Health centers primarily serve outpatients, except in the case of maternity where women and new-borns may stay 24 to 72 h [18]. Caregivers, such as the mothers of pediatric patients and family members of women in maternity, are also regular visitors at HCFs and may stay overnight.

National evaluations conducted by the Ministry of Infrastructure of Rwanda in 2009 found that 37% of health centers in Rwanda had piped water and 29% had a piped water within the facility [11, 19]. A national survey of HCFs in Rwanda (World Bank Service Provision Assessment 2007) found that 28% of health centers in Rwanda had *year-round* water supplied in the facility by tap or available within 500 m of the facility. The survey reported that 59% of health centers had power routinely available during service hours or a backup generator with fuel, and 58% had a functional client latrine, a waiting area protected from sun and rain, and basic levels of cleanliness [20]. Rwanda has a national strategic plan for management of healthcare waste and has demonstrated better access to infection control materials in HCFs than other countries in Africa [5].

The main objectives of this research were: 1) to conduct systematic, rapid screening assessments of HCF in Rwanda to assess the need and suitability of the HCF to receive a donation of a water treatment system (WTS), and 2) to conduct baseline assessments of WASH in the HCF where WTS were to be donated in order to measure the effects of the WTS intervention on water quality and WASH services in the HCF. This study describes the state of WASH in rural secondary HCFs in Rwanda utilizing data from the systematic rapid screening assessments and baseline assessments. The authors describe the performance and suitability of the WTS elsewhere [21]. The WTS used membrane ultrafiltration and chlorination for water purification and required reliable sources of water and power to function. Therefore, the data described here represent HCFs with better access to piped water and above average infrastructure, as compared to other HCFs in Rwanda and in the Sub-Saharan Africa region overall [22, 23]. This study goes beyond simple measures of infrastructure at the facility level, as provided in nationally representative service delivery assessments [24, 25], and examines the provision of improved WASH services for all of the multiple populations in these HCFs: patients, caregivers and HCF staff.

## Methods

### Site selection

The HCFs included in this assessment were selected in collaboration with researchers at the Rwanda Ministry of Health, the Center for Global Safe Water, Sanitation and Hygiene at Emory University, and the Access Project Rwanda. Sites were selected for their potential to participate in a two-year feasibility assessment of advanced on-site WTS donated to ten HCFs. Minimum eligibility criteria were HCF location within two predetermined districts in Rwanda where the partner organizations operated, access to an improved on-site water source, a reliable source of power, and willingness of the HCF director to receive the donated WTS and participate in the research study.

### Data collection and analysis

Data were collected at 17 HCF in total over two rounds of assessments. A rapid screening assessment was conducted in 15 HCF in May–June 2011 in order to assess the suitability of donating a WTS to the HCF. Following the rapid screening assessment, project partners and beneficiaries convened in order to agree upon which HCFs would receive a WTS. Among the 15 HCFs included in the rapid screening assessment, two HCFs were excluded due to inadequate piped water infrastructure to support a WTS, two HCFs opted out of the donation program because the HCF director did not want a WTS, and three HCFs were excluded because of their geographic isolation, leaving eight participating HCFs. In order to donate a total of ten WTS, two additional HCFs meeting the inclusion criteria were added, resulting in a total of 17 sites included in this study. Due to study constraints, a rapid screening assessment was not done at the two HCFs added after the first round of data collection. In December 2012 a baseline assessment was conducted at the ten HCF where the WTS were going to be installed. Data were collected during unannounced visits by trained research staff from Emory University and The Access Project Rwanda. Both rounds of data collection took place during dry season in Rwanda.

### Rapid screening assessment

In May–June 2011, a rapid assessment of WASH and environmental services in the HCFs was conducted at 15 health centers that were recommended by the Ministry of Health as potential candidates for receiving WTS. This assessment included direct observations of the HCFs’ infrastructure for water and power, a semi-structured interview with the director of the HCF, and collection of tap water and treated drinking water (if available) from the most frequently used taps or containers.

### Baseline assessment

In December 2012, a baseline assessment was conducted at the ten HCFs chosen to receive the WTS. Baseline assessments included sampling tap water and treated drinking water (if available) from the most frequently used taps or containers, and interviews with HCF staff responsible for facility management, including WASH, that addressed water and power; drinking water availability and treatment; cleaning, maintenance, and repair of WASH infrastructure; and WASH-related record keeping. A systematic inspection of the water distribution network, rainwater catchment system, hand washing infrastructure and toilets and latrines was conducted. Toilets and latrines were inspected for functionality and hygienic condition, and HCF staff indicated whether facilities were for use by staff or patients and visitors. Toilet and latrine observations were conducted using a protocol developed by Emory University for previous evaluations of school WASH. Observations about type of sanitation facility and visible feces, flies and odor were made independently by two raters. Data were recorded on paper surveys and then entered and analyzed in Microsoft Excel (Redmond, WA, USA).

### Water sample analysis

The same water sampling and analysis methods were applied in both the 2011 and 2012 rounds of data collection. A study manager from Emory University research directly supervised all data collection and water sampling and testing. Water samples were collected in duplicate using 100 mL pre-sterilized sample collection bags (Nasco, Fort Worth, TX, USA). Sodium thiosulphate was used for chlorinated water to preserve samples for microbiological analysis. Samples were transported on ice to the district hospital for analysis within 4 h of collection. Undiluted 100 mL water samples were analyzed for total coliforms and *E. coli* using the IDEXX Quanti-Tray/2000 system with Colilert reagent according to the manufacturer’s directions (IDEXX, Westbrook, ME, USA). Microbial concentrations were estimated using the Most Probable Number (MPN) method provided by the manufacturer. Limits of detection were 1 to 2419.6 coliforms or *E. coli* per 100 mL. Duplicate samples were analyzed for total and free chlorine residuals (N,N-diethyl-p-phenylenediamine (DPD) method, digital colorimeter, HACH, Loveland CO, USA).

## Results

### Water supply and power

On the day of assessment, all HCFs had power and 15 of 17 had running water on site, with plumbing that reached each service of the HCF. Twelve HCFs had power provided by the national utility and five HCFs had working solar panels. Eleven HCFs had piped water on site supplied by the national utility (treated surface water). The remaining six HCFs had piped water on site from a local water source (untreated water from protected and unprotected springs) (Table 1). Nine of the 17 HCFs were located in the Eastern Province, and eight were in the Northern Province. Staff at the HCFs located in the Eastern Province reported that seasonal water shortages were common during the dry season – approximately June to September. In the Northern and Western Provinces, where annual rainfall typically exceeds 1200 mm/year [26], water shortages were reported to be infrequent (no more than once per month and less than 1 day in duration). Fifteen of 17 HCFs had rainwater catchment systems that ranged in volume from 5 m^3^ to 100 m^3^ (Table 1).

**Table 1:** General water and power characteristics of 17 rural healthcare facilities in Rwanda, assessments conducted in 2011 and 2012

Health center	Power source	Primary water source^a^	Rainwater storage	Drinking water treatment method^b^	Assessments conducted^c^
A	Solar	National utility	100 m^3^	Ceramic filter	Rapid 2011, Baseline 2012
B	Grid	National utility	30 m^3^	Boiling	Rapid 2011, Baseline 2012
C	Grid	National utility	13 m^3^	Ceramic filter	Rapid 2011, Baseline 2012
D	Grid	National utility	100 m^3^	POU chlorination	Rapid 2011, Baseline 2012
E	Grid	Local supply (untreated)	11 m^3^	POU chlorination	Baseline 2012
F	Grid	National utility	20 m^3^	POU chlorination and ceramic filter	Baseline 2012
G	Solar	Local supply (untreated)	55 m^3^	POU chlorination and ceramic filter	Rapid 2011, Baseline 2012
H	Solar	National utility	30 m^3^	POU chlorination and ceramic filter	Rapid 2011, Baseline 2012
I	Grid	National utility	20 m^3^	POU chlorination and ceramic filter	Rapid 2011, Baseline 2012
J	Grid	Local supply (untreated)	20 m^3^	POU chlorination	Rapid 2011, Baseline 2012
K	Grid	Local supply (untreated)	10 m^3^	POU chlorination	Rapid 2011
L	Grid	National utility	10 m^3^	POU chlorination	Rapid 2011
M	Grid	National utility	10 m^3^	POU chlorination	Rapid 2011
N	Grid	National utility	5 m^3^	No treatment	Rapid 2011
O	Grid	National utility	none	Ceramic filter	Rapid 2011
P	Solar	Local supply (untreated)	15 m^3^	No treatment	Rapid 2011
Q	Solar	Local supply (untreated)	none	Ceramic filter	Rapid 2011

^a^Piped water supplied by the national utility Water and Sanitation Corporation (WASAC) was surface water treated at centralized facilities, local piped water supply was untreated water piped from protected and unprotected springs. ^b^Ceramic filters had two or four candles and 8 L capacity; point of use (POU) chlorination was done in 20 L containers using dilute sodium hypochlorite solution. ^c^ Rapid screening assessments consisted of: observation of HCF infrastructure; interview with HCF director; water sample collection and analysis. Baseline assessments consisted of: interviews with HCF staff responsible for facility management, drinking water treatment, cleaning, maintenance and repair of WASH infrastructure, WASH-related record keeping; systematic inspection of WASH infrastructure including latrines and toilets; water sample collection and analysis

### Drinking water treatment

Staff responsible for WASH at 15 of the 17 HCFs (88%) reported using some type of point-of-use water treatment method for drinking water. Piped water was reported as the primary source of water treated for drinking at all sites. When this source was unavailable, HCF staff reported treating rainwater for drinking. At six of 17 HCFs (35%) chlorine solution (SûrEau brand) was used for drinking water treatment, and ceramic filters (2 or 4 filters, 8 L capacity) were used at four of 17 HCF for drinking water treatment. At four of 17 HCFs (24%) both chlorine solution and ceramic filters were used. At one of 17 HCFs (6%) staff reported boiling water, and staff at two of 17 HCFs (12%) indicated that water was not treated before provision for drinking. The eight HCFs included in both the rapid and baseline evaluations had the same responses for drinking water treatment practices in both 2011 and 2012. (Table 1).

Staff responsible for WASH at all 17 HCF indicated that drinking water, in general, was available to anyone who wanted it. However, at all sites where drinking water was available on the day of assessment (ten sites during rapid screening assessments and eight during baseline assessments) it was observed that water was treated in the pharmacy via ceramic filters or addition of chlorine solution, and treated drinking water was available in limited quantities of up to 20 L per day.

### Water quality

In the rapid screening assessment conducted in May–June 2011, tap water was available to sample at 14 of 15 HCFs, and drinking water was available to sample at 10 of 15 HCFs.

**Fig. 1:**
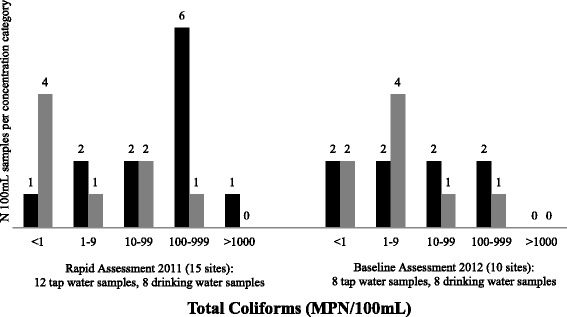
Total coliform concentration (MPN/100 mL) in tap water samples and treated drinking water samples from rural health centers in Rwanda collected during rapid assessment in 2011 and baseline assessment in 2012. *Black:* Tap water samples, *Grey:* Treated drinking water samples. Rapid assessment results for 12 tap water samples and 8 drinking water samples are presented. Note that 1HCF had no water and 4 HCFs had no drinking water at the time of the assessment visit. Samples from 2 HCF were excluded because the amount of time between sample collection and analysis exceeded the time defined in standard methods. Baseline assessment results for 8 tap water samples and 8 drinking water samples are presented. No water was available at 2 HCF during the assessment visit

**Fig. 2:**
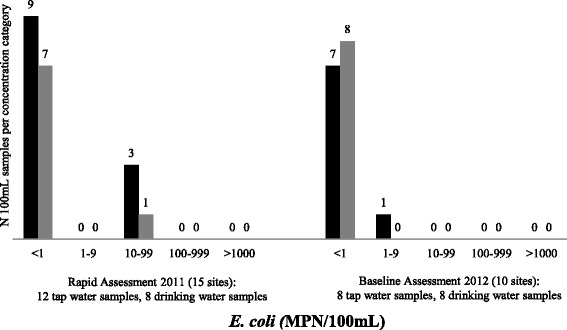
*E. coli* concentration (MPN/100 mL) in tap water samples and treated drinking water samples from rural health centers in Rwanda collected during rapid assessment in 2011 and baseline assessment in 2012. *Black:* Tap water samples, *Grey:* Treated drinking water samples. Rapid assessment results for 12 tap water samples and 8 drinking water samples are presented. Note that 1 HCF had no water and 4 HCFs had no drinking water at the time of the assessment visit. Samples from 2 HCF were excluded because the amount of time between sample collection and analysis exceeded the time defined in standard methods. Baseline assessment results for 8 tap water samples and 8 drinking water samples are presented. No water was available at 2 HCF during the assessment

**Fig. 3:**
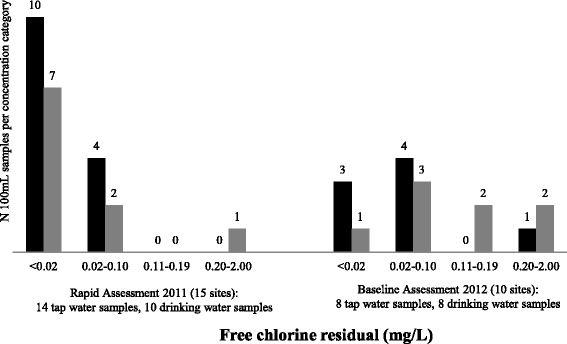
Free chlorine residual (mg/L) in tap water samples and treated drinking water samples from rural healthcare facilities in Rwanda collected during rapid assessment in 2011 and baseline assessment in 2012. *Black:* Tap water samples, *Grey:* Treated drinking water samples. Rapid assessment results for 14 tap water samples and 10 drinking water samples are presented. Note that 1 HCF had no water and 4 HCFs had no drinking water at the time of the assessment visit. Baseline assessment results for 8 tap water samples and 8 drinking water samples are presented. No water was available at 2 HCF during the assessment

The results from 14 tap water samples and 10 treated drinking water samples are included in the final data analysis for chlorine residual. The microbiological results for two tap water samples and two treated drinking water samples were excluded from the final analysis because the amount of time between sample collection and analysis exceeded the time defined in standard methods. One of 12 tap water samples (8%) and four of eight treated water samples (50%) met the WHO guideline of <1 total coliform per 100 mL (Fig. 1). Nine of 12 tap water samples (75%) and 7 of 8 treated drinking water samples (88%) met the WHO guideline of <1 *E. coli* per 100 mL (Fig. 2). No tap water sample (0 of 14) and only one of ten treated drinking water samples (10%) met the WHO guideline of free chlorine residual ≥0.2 mg/L (Fig. 3) despite the fact that staff at 10 of 14 HCFs with water available reported treatment of drinking water with chlorine solution (Table 1).

In the baseline assessment conducted in December 2012, tap water and drinking water were available to sample at eight of 10 HCFs. Two of eight tap water samples (25%) and two of eight treated water samples (25%) met the WHO guideline of <1 total coliform per 100 mL (Fig. 1). Seven of eight tap water samples (88%) and all treated drinking water samples (8 of 8) met the WHO guideline of <1 *E. coli* per 100 mL (Fig. 2). One of eight tap water samples (13%) and two of eight treated drinking water samples (25%) met the WHO guideline of free chlorine residual ≥0.2 mg/L (Fig. 3) even though staff at seven of eight HCFs with water available reported using chlorine solution (Table 1).

### Water access within the healthcare facility

Baseline assessments were conducted in December 2012 at 10 sites. Each HCF assessed had several types of water access points: there were 181 sinks with taps within the HCFs’ services and at toilets; the number ranged from six to 30 per site (Table 2). The number of sinks at each HCF was in part determined by the number of different buildings housing the various services at the HCF and the extent of plumbing to each building. There were 33 tippy taps (non-networked hand washing locations with a water container and a tap) located primarily at latrines (ranging from one to eight per HCF). Outdoors, in common areas of the HCFs, there were 44 rainwater tanks with taps (ranging from one to eight per HCF) and a small number of outdoor taps (nine outdoor taps across six HCFs) (Table 2).

**Table 2:** Functionality of water access points in 10 rural healthcare facilities in Rwanda, December 2012

Health Center	Sinks with taps^a^ N functional / Total	Outdoor taps N functional / Total	Tippy taps^b^ N functional / Total	Rainwater tanks with taps N functional / Total	All water access points N functional / Total (%)
A	11 / 19	0 / 0	1 / 1	0 / 1	12 / 21 (57)
B	19 / 24	0 / 0	1 / 4	3 / 4	23 / 32 (72)
C	7 / 8	0 / 0	3 / 4	0 / 2	10 / 14 (71)
D	17 / 21	0 / 0	1 / 4	0 / 1	18 / 26 (69)
E	10 / 10	0 / 2	7 / 8	3 / 5	20 / 25 (80)
F	25 /30	1 / 1	2 / 2	3 / 6	31 / 39 (79)
G	4 / 16	0 / 1	3 / 3	3 / 6	10 / 26 (38)
H	16 / 28	0 / 2	1 / 2	2 / 8	19 / 40 (48)
I	4 / 6	0 / 1	0 / 3	0 / 5	4 / 15 (27)
J	6 / 19	2 / 2	1 / 2	4 / 6	13 / 29 (45)
TOTAL (% Functional)	119 / 181 (66)	3 / 9 (33)	20 / 33 (61)	18 / 44 (41)	160 / 267 (60)

^a^Sinks with taps were located within HCF laboratory, pharmacy, administration, consultation, hospitalization and maternity services, and at toilets. ^b^Tippy taps were non-networked hand washing locations with a water container and a tap, located primarily at latrines

At all HCFs, certain sinks with taps were dedicated for staff use only, including those in the laboratory, pharmacy, administration, consultation, and maternity services. There were sinks with taps in the hospitalization service wards for inpatients at all HCFs. There were sinks with taps located at toilets, and tippy taps at latrines, that were accessible to patients and visitors unless the toilet/latrine was dedicated for staff use only. The water access points located in outdoor common areas that were accessible to patients and visitors were outdoor taps and rainwater tanks with taps. In total, 40% of the water access points were not functioning on the day of inspection (Table 2). One third of taps at sinks were in various states of disrepair; with the predominant problem being leaks that were managed by turning off the water supply to the tap. Lack of water elsewhere was due to empty tippy taps and lack of handles on outdoor taps and rainwater tank taps. Staff responsible for WASH at four of the ten HCF reported that they had intentionally removed the handles as a measure to manage water use.

### Hand hygiene infrastructure

Available hand hygiene infrastructure (water access points used for hand washing) included sinks with taps, outdoor taps, and tippy taps. Rainwater tanks with taps were not identified by HCF staff as part of hand hygiene infrastructure and were therefore excluded. Sinks with taps were the most common form of hand hygiene infrastructure; there were 119 working sinks with taps, ranging from four to 25 per HCF (Table 2). Soap was provided at 39 of the 119 working sinks with taps (33%) ranging from zero (0%) to 12 (75%) per HCF (Table 3). Provision of soap at tippy taps was observed at two out of ten HCFs. No soap was provided at outdoor taps at any HCF. The availability of soap ranged from no soap at any hand washing locations in the HCF, to soap at 12 out of 17 (71%) hand washing locations (Table 3).

**Table 3:** Availability of soap and water for hand washing in 10 rural healthcare facilities in Rwanda, 2012

Health center	Number of sinks with taps with soap and water (% of total with water)^b^	Number of tippy taps with soap and water (% of total with water)^b^	Total number of hand wash locations with soap and water (% of total with water)^a^
A	1 (9)	0 (0)	1 (8)
B	5 (26)	0 (0)	5 (25)
C	3 (43)	0 (0)	3 (30)
D	3 (18)	0 (0)	3 (17)
E	5 (50)	5 (71)	10 (59)
F	5 (20)	2 (100)	7 (25)
G	0 (0)	0 (0)	0 (0)
H	12 (75)	0 (0)	12 (71)
I	1 (25)	0 (0)	1 (25)
J	4 (67)	0 (0)	4 (44)
TOTAL	39 (33)	7 (35)	46 (32)

^a^Hand wash locations included sinks with taps, tippy taps (non-networked hand washing locations with a water container and a tap), and outdoor taps; no soap was available from any outdoor tap, therefore outdoor taps are not included in this table

^b^Percentage of hand wash locations with soap and water available were calculated using the number of hand wash locations with soap divided by the number of functional water access points from Table 2

Although soap was observed at few hand washing locations, nine out of ten HCF directors reported that there were sufficient funds to purchase soap for hand washing, The HCF staff responsible for WASH at six of ten HCF reported that it was difficult to provide soap because of misuse or theft by the HCF visitors. Provision of hand sanitizing rub was not observed at any HCF.

### Toilets and latrines

All HCFs assessed had toilets and/or latrines for staff, patients and visitors. Toilets were flush toilets with pedestals and squat plates; latrines were pit latrines and improved ventilated pit latrines. There were a total of 47 toilets and 62 latrines at the ten HCF, ranging from 6 to 15 toilets and/or latrines per site (Table 4). Two thirds of toilets were in use, and 53% of latrines were in use and in hygienic condition (defined as absence of two or more of the following characteristics: visible feces, flies, strong odor before entering the facility). The average combined proportion of toilets and latrines that were in use and in hygienic condition per HCF was 59% and ranged from 25 to 100% per site (Table 4). The majority, 30 out of 33 (91%), of latrines that were in use and in hygienic condition were accessible for use by patients and visitors. At nine out of ten HCFs, at least one flush toilet was dedicated for staff use only and was usually kept locked; among the toilets at HCFs that were in use and in hygienic condition, 18 out of 31 (58%) were available to patients and visitors and the remaining 13 out of 31 (42%) were reserved for staff only (Table 4).

**Table 4:** State of toilets and latrines in 10 rural healthcare facilities in Rwanda, 2012

Health center	N toilets and latrines combined	n toilets, n latrines^a^	n toilets, n latrines in use, in hygienic condition (% combined)^a^	n toilets, n latrines in use in hygienic condition^b^ and accessible^c^ to patients and caregivers (% combined)^d^
A	11	7, 4	4, 0 (36)	3, 0 (27)
B	12	4, 8	2, 1 (25)	2, 0 (17)
C	10	2, 8	2, 3 (50)	0, 3 (30)
D	15	11, 4	4, 0 (27)	2, 0 (13)
E	12	3, 9	2, 9 (92)	2, 9 (92)
F	8	2, 6	1, 6 (88)	0, 4 (50)
G	11	2, 9	2, 9 (100)	0, 9 (82)
H	11	3, 8	3, 1 (36)	2, 1 (27)
I	6	6, 0	6, 0 (100)	4, 0 (67)
J	13	7, 6	5, 4 (69)	3, 4 (54)
TOTAL	109	47, 62	31, 33 (59)	18, 30 (44)

^a^Toilets were flush toilets with pedestals and squat plates; latrines were pit latrines and improved ventilated pit latrines. ^b^Absence of 2 or more of the following: odor, flies, and feces. ^c^Accessible was defined as unlocked and designated by HCF staff for use by patients and caregivers

^d^Combined percentages of toilets and latrines in use, in hygienic condition and available to patients and caregivers were calculated using the number of toilets and latrines combined, divided by the number accessible

### HCF staff roles and responsibilities

Core, non-clinical staff at the HCFs included Environmental Health Officers (EHOs) among whose responsibilities are infection control, waste disposal and WASH. In 2013, the Ministry of Health reported 227 EHOs: approximately one EHO for every two HCF and one EHO per 48,000 people [11]. HCFs in Rwanda have at least one maintenance worker, a hygienist, who is responsible for cleaning the facility and managing water use. Under supervision from the EHO and clinical staff, hygienists treat drinking water, provide soap and water for hand washing, clean toilets and latrines, and manage HCF waste disposal. Among the HCFs evaluated, there was one EHO and two or more hygienists at each site. EHOs at all HCFs reported that 20 L of treated drinking water were prepared each day and that latrines and toilets were cleaned at least once per day. These activities were not documented in writing in a register or other format, rather, EHOs reported that they managed these activities and reported problems to the director when necessary. Roofs and rain gutters were reported to be cleaned once per year. None of the HCFs had on-site capacity or basic tools for servicing electrical systems or plumbing: any electrical or plumbing repairs, such as leaking taps, were contracted to outside technicians. HCFs used local private services to empty latrine pits. Distribution of toilet paper in patient/public use sanitation facilities was limited due to concern about misuse or theft. A commonly reported sanitation maintenance problem was blocked toilets and pipes.

### Record keeping and reporting

Record keeping associated with WASH infrastructure was limited to a requisition form for soap, toilet paper, and detergent that was managed by HCF accountants. No records were kept of water availability or drinking water treatment, nor sanitary facility cleaning or repair. HCF accountants were responsible for procuring water treatment products, soap, toilet paper, and other cleaning agents for HCF hygiene, and paying utility bills. No HCF reported that they had outstanding water or power bills.

There was external monitoring of water and sanitation at the HCF through the Government of Rwanda Performance-Based Financing System (PBF). At the time of data collection, the PBF evaluation was conducted quarterly and included observation of on-site piped water and drinking water in the pharmacy. District-level oversight systems also included observation of whether there was a ceramic filter for water treatment in the pharmacy [27]. District-level evaluations were conducted at the same time as the PBF evaluation. These evaluations were conducted by team of District-level hospital staff including District EHOs and results were reported to the Ministry of Health at District and central levels.

## Discussion

Multi-country reports by WHO and analyses of service delivery assessments have called attention to the disparities in provision of WASH services in the African region, particularly in rural secondary healthcare facilities. Among 52,000 HCFs in 23 countries across Africa, just over half had an improved water source within 500 m [5, 9, 10]. Beyond these sobering figures, the reports emphasize that few low-income countries have policies and plans for sanitation, hygiene and drinking water in HCFs [10, 28]. Rwanda is among 16 African countries that were able to demonstrate that plans for water provision in HCFs have been developed and are being implemented [10]. The HCFs included in this evaluation are not representative of rural secondary HCFs across Africa; both in terms of infrastructure and manpower they are better equipped [8, 14, 18]. However, the findings of this small, in-depth, evaluation offer evidence that standard service delivery indicators do not accurately reflect access to improved WASH by staff, patients, and caregivers. This study offers an example of improved infrastructure and dedicated personnel succeeding in meeting WASH provision standards where an evaluation framework is in place, as evidenced by the provision of treated water in the HCF pharmacies following Rwanda’s PBF system.

### Water supply

All the HCFs evaluated had on-site access to water. This is a major improvement upon the infrastructure norms of secondary HCFs in rural sub-Saharan Africa [5, 7, 14]. However, on-site water access at HCFs did not directly translate into water access for patients and visitors. There were 119 functioning sinks with taps (66% of 181 total) primarily located within HCF services, but a combined total of 41 functional tippy taps, outdoor taps and rainwater tanks that were readily accessible to patients and visitors in the common areas of the HCFs.

### Drinking water

WHO guidelines recommend ‘a reliable drinking-water point accessible for staff, patients, and caregivers at all times’ [1]. Staff at the HCFs reported treating up to 20 L of drinking water per day. With HCF receiving approximately 100 outpatients per day, this volume of safe drinking water equated to only 200 mL per outpatient per day and did not take into account the special needs of in-patients in maternity, feeding programs for child nutrition, caregivers or staff. Furthermore, treated drinking water was only available in the pharmacy of each HCF. In the 17 HCFs evaluated, less than 20% of treated drinking water samples met WHO guidelines for total coliforms and free chlorine residual, but just one of 17 samples had ≥1 *E. coli* per 100 mL.

### Hand hygiene infrastructure

The WHO recommends that HCFs have ‘a reliable water point, with soap or a suitable alternative, available at all critical points within the health-care setting and in service areas’ and ‘at least two hand washing sinks should be provided in wards with more than 20 beds’ [1]. The HCFs evaluated had, on average, 20 beds per facility (in maternity and hospitalization services) and four separate toilet/latrine blocks. Critical hand hygiene locations were the waiting area, consultation, outpatient care, maternity, hospitalization, pharmacy, laboratory, voluntary counseling and testing, antenatal care, vaccination/nutrition, and at toilets/latrines. Taking into account that at these small HCFs some services shared a sink due to close proximity, seven out of ten HCFs evaluated had water available at all critical hand hygiene locations, and overall, almost one third of hand wash locations had soap available. Consistent with observations addressing the availability of water, most of the water access points with soap were found in areas for staff members, not patients or caregivers.

### Toilets and latrines

The WHO guideline for excreta disposal in HCFs recommends that ‘there are sufficient toilets available: one per 20 users for inpatient settings; at least four toilets per outpatient setting (one for staff, and for patients: one for females, one for males and one for children)’ [1]. All HCFs had sufficient sanitary infrastructure to meet and exceed this recommendation, however, if the accessibility and hygienic state of the sanitary facilities are considered, then five of the ten HCFs met the guideline (five HCFs had <4 toilets/latrines in hygienic state and accessible to patients and caregivers).

### Impact on health service delivery

Limited access to clean and reliable water supplies has a direct impact on quality of care and maternal and child survival [29]. Rural HCFs providing maternity services may have no water at all or may expose women to unsafe water [5, 30]. Poor WASH provision at HCFs deters medical care-seeking behavior at HCFs. A qualitative and observational study of early discharge from HCFs in Tanzania following childbirth found that lack of access to water for post-delivery bathing and for guests/caretakers to prepare meals at the hospital was a commonly reported reason for leaving facilities less than 24 h after giving birth [31]. A review of WASH and maternal child health emphasized that in the few published examples available, poor and inadequate sanitation in HCFs was a major cause of dissatisfaction among patients, and women may avoid seeking institutional delivery care because of lack of toilets [32]. Even in this study, which focused on facilities with robust infrastructure, less than half of the sanitation facilities for patients and visitors were in a hygienic state.

The growing body of literature on WASH and maternal child health in HCFs advocates for nationally-mandated indicators, ministerial initiatives, internal regulation, and independent quality control for WASH in HCFs as essential steps in improving healthcare delivery services [9, 29, 30, 32]. Studies such as the assessment presented here offer evidence about actual conditions and the need for WASH indicators that go beyond facility-level infrastructure and include actual routine provision of services. While policies and plans for WASH in HCFs continue to be developed, Rwanda has recently codified detailed indicators of WASH services provision (such as water quality, quantity, and availability) into a nationally–adopted hospital accreditation evaluation program administered by the Council for Health Service Accreditation of Southern Africa [33]. This advance demonstrates financial commitment and policy integration towards internal regulation and independent quality control for WASH in HCFs.

### Strengths and limitations

Strengths of this assessment include: observation as opposed to self-reported survey data, systematic objective observations made by trained researchers under direct supervision of the study manager and conducted during unannounced visits, and research into national and sub-national policy. The selection of sites was non-random and not representative of all HCFs in Rwanda. This study does not offer information on changes in WASH infrastructure and water availability over time, the use of WASH infrastructure by staff, patients, and caregivers, nor does it address waste management or disinfection practices, which are critical aspects of infection control. Data were collected in 2011–2012, HCF infrastructure and HCF staff practices for provision of WASH services may have changed between 2012 and 2017. While not extensible, this analysis and contextualization within national and sub-national policies offers detail that is otherwise unavailable on the monitoring and evaluation systems and prioritization in provision of WASH services for staff, patients, and caregivers at HCFs.

## Conclusions

At the HCFs evaluated, indicators for water access were regularly monitored in national and sub-national plans, and these indicators had better performance than other aspects of WASH in HCF addressed in WHO guidelines. This underscores the importance of internal and external monitoring of WASH in HCFs. This study provides evidence that water “access” may not equate to sufficient water quantity and quality by WHO guidelines. Direct observation of WASH facilities allowed assessment of the functionality of water and sanitation infrastructure, which is not captured in assessments of “access” which may overestimate WASH coverage in HCFs. While good WASH practices by clinical staff are essential for infection control and prevention, this study found that there was limited access to WASH infrastructure for patients and caregivers. This is relevant to the development of national policies and plans in settings where the ratio of trained medical professionals to patients is low and caregivers provide most of the daily supportive care. Further investigation is needed to address the enabling factors and constraints for provision, use, and maintenance of WASH infrastructure at HCFs.

## Abbreviations

DPD N: N-diethyl-p-phenylenediamine
*E. coli*: *Escherichia coli*
EHO: Environmental Health Officer
HAI: Hospital-acquired infection
HCF: Healthcare facility
MPN: Most Probable Number
PBF: Performance-based Financing
POU: Point of use
WASAC: Water and Sanitation Corporation
WASH: Water, sanitation and hygiene
WHO: World Health Organization
WTS: Water Treatment System
